# Correction: “Crises are a perpetual restart” – a comparative analysis of maternal and newborn health political prioritization across four fragile and conflict affected settings

**DOI:** 10.1371/journal.pgph.0006214

**Published:** 2026-03-31

**Authors:** The PLOS One Staff

## Notice of Republication

This article was republished on March 17, 2026, to correct an error in the author list. Naoko Kozuki was erroneously displayed as Naoko Kozuk. The publisher apologizes for the error. Please download this article again to view the correct version. The originally published, uncorrected article and the republished, corrected articles are provided here for reference.

## Supporting information

S1 File Originally published, uncorrected article. (PDF)

S2 File Republished, corrected article. (PDF)
