# Supplementary material for: “Crises are a perpetual restart”—A comparative analysis of maternal and newborn health political prioritization across four fragile and conflict affected settings
Source: PLOS Glob Public Health. 2025 Oct 22;5(10):e0005288. doi: 10.1371/journal.pgph.0005288 (PMC12543140; doi:10.1371/journal.pgph.0005288)
Supplement: Attachment — Submitted filename: Response to Reviewers_final.docx [file pgph.0005288.s003.docx]

Dear Editor,

Thank you for your feedback on our manuscript, and many thanks to the reviewers for their comments, which have helped to improve its quality.

Please note that on the request to share data, we request exemption to sharing data in a public repository. Our data, which discusses political issues in policymaking and implementation, cannot be made publicly available out of concerns of the inability to sufficiently deanonymize the data.  The original studies entail key informants at the national/state level, with potentially very high possibility of identification since there are only so many people who have leadership roles in the maternal and newborn health space in these geographies.

We appreciate any feedback or advice on how to handle the data,

Please see below a table of our responses related to each comment by reviewers.

| **Comment** | **Response** |
| --- | --- |
| ***Editors’ comments*** | |
| We have noticed that you have a list of Supporting Information legends in your manuscript. However, there are no corresponding files uploaded to the submission. Please upload them as separate files with the item type 'Supporting Information'. | We have reviewed our submission and the supporting information stated in the manuscript are   - *S1 Table 2 Comparison of country cases across political and socioeconomic dimensions* - *S2 Table 4: Summary of key sub-themes emerging from analysis of MNH prioritization factors across categories of the Shiffman and Smith (2007) framework*   These were included as part of the submission as Supplemental. We have now re-attached them to the submission under Supporting Information. |
| In the online submission form, you indicated that “Data can be accessed from the manuscript authors.”.    All PLOS journals now require all data underlying the findings described in their manuscript to be freely available to other researchers, either  1. In a public repository,  2. Within the manuscript itself, or  3. Uploaded as supplementary information.    This policy applies to all data except where public deposition would breach compliance with the protocol approved by your research ethics board. If your data cannot be made publicly available for ethical or legal reasons (e.g., public availability would compromise patient privacy), please explain your reasons by return email and your exemption request will be escalated to the editor for approval. Your exemption request will be handled independently and will not hold up the peer review process, but will need to be resolved should your manuscript be accepted for publication. One of the Editorial team will then be in touch if there are any issues. | Thank you. Our data cannot be made publicly available for ethical reasons and we have written to the journal to request exemption, with the commitment to share anonymized data on a case by case basis. We include this request in this letter too as per journal feedback to our email. |
| Please review your reference list to ensure that it is complete and correct. If you have cited papers that have been retracted, please include the rationale for doing so in the manuscript text, or remove these references and replace them with relevant current references. Any changes to the reference list should be mentioned in the rebuttal letter that accompanies your revised manuscript. If you need to cite a retracted article, indicate the article’s retracted status in the References list and also include a citation and full reference for the retraction notice. | Noted |
| ***Reviewer 1*** | |
| Thank you for the opportunity to review this insightful paper on the political prioritization of MNH in fragile and conflict affected settings. It is powerful, valuable and timely research. | Thank you very much. |
| Overall, there is substantial relevant data to show the various factors shaping MNH prioritization in these settings. In the Findings, there are some really strong sections presenting clear arguments that respond to the research question, such as the “effective, system-wide interventions” section. However, there are other times when there are several different points being made at once, which makes it difficult to follow the thread of the argument, or understand which are the most salient issues regarding prioritization. Some paragraphs start with a focus on one issue, then jump to another – e.g. the paragraph starting at Line 286, starting with discussion of political leadership and then shifting to environmental/health crises. There are a few other places where there are a few connected ideas, but the link is not made explicitly or the overall point fleshed out, such as with paragraphs starting Line 226 and Line 368 (for instance with the Line 368 paragraph, how does religious representation influenced issue framing and what is its impact on prioritization?). Perhaps some of these are not such significant factors in political prioritization – it would be interesting to know what the data suggests is really key and has consensus within/across informants, versus those with less explanatory power.  Finally for the Results, it could be made clearer at times how the different points link to political prioritization specifically, rather than governance generally. | The line on political manifesto in 286 (now line 303) has been joined to the paragraph before which talks about political developments as policy windows. Then the crises as policy windows paragraph becomes much clearer from line 305, where we have added an introductory sentence to ensure this shift is clear – “Besides political events, health emergencies and other crises have also served as policy windows to generate more investments in MNH”. Then it follows with examples from Somalia and DRC. Thank you for pointing this out.  We have improved the link between line 226 and the succeeding sentences by adding “. Besides research and evidence, there are other factors that drive prioritization by governments and NGOs”. Please see lines 246 to 250. This sets up the mention of public outcry and maximization of beneficiary numbers in the sentences that follow. Thank you for pointing this out.  In the paragraph starting in line 368, now line 386, we have elaborated on issue framing and its relationship to religion. The way the actors understand and portray the issue can be influenced by their religion among other factors. While there might synergies between Islam and Christianity on the importance of MNH, health service expectations and preferences may differ based on different restrictions around childbirth and women’s participation in public life. Thus if representation is unequal in decision making tables, it means preferences of Christian patients may not be represented. This explanation has now been included in lines 390 to 394, with some references also added.  In general, these factors are important for political prioritization as contextual factors and influences on ideas. Our analysis focused on key issues that informed the framework, and we hope these additional descriptions and citations improve the coherence of the arguments and relate them more directly to political prioritization as per the framework.  We have reviewed the Results section and made the following additional edits to link to political prioritization framework specifically:   - Line 230 to 232: linking research and evidence to credible indicators, which is a subtheme to understanding issue characteristics or the features of high maternal and newborn morbidity and mortality as a problem. - The section on policy windows: we have clarified with description and included references on how health emergencies are not just a governance issue but political as well, as well as their linkage. Please see lines 305 to 308. - The section on governance structures, please see lines 335 to 337 linking governance to actor power, interests, collaboration. This links the rest of the section on territorialization, poor coordination and poor local ownership to these underlying political drivers. The next section on Actor Power from line 364 then delves into these further. - From line 419 on women leadership, please see line 423-424 adding political representation in legislative and other decision-making bodies; the inclusion of women as a political choice (lines 428-429, and lines 433-434) - Guiding institutions: this relates to power of institutions to lead MNH agenda, beyond just technical governance. We have fixed paragraph 476 to 486 to read clear, and emphasized how power is related to the ability of actors especially government to coordinate partners. - Grammatical edits under Policy Community Cohesion - Ideas: this section relates broadly to stakeholder perception of MNH as priority, and we did not have enough data to explore relationship to political ideas or external framing by the public, which was out of scope of study. We made minor grammatical edits. |
| In the Introduction, I appreciate the comprehensive context provided, but it could at times be focused more tightly on MNH policy, its impact and why political prioritization is important to understand/ analyze (especially paragraphs 2 & 3). | In paragraph 2, we have added lines 75 to 83 with new references elaborating on how fragility is linked to MNH policy, and how political prioritization fits in. In paragraph 3, we have made the more direct link between governance and political will as it impacts health. We also link political will and prioritization of MNH to improved outcomes via increased coverage and uptake of services (lines 93 to 95). |
| In the Methodology section – why are some data national and some state level? What impact does this have on findings?  I appreciate the explanation of how the framework is being applied to the case, and am curious whether the findings diverged from the framework in any way (other than not including external framing for practical reasons)? So, for example, if any key categories emerged that could not fit into the framework. | The focus of the study was on fragile contexts; some countries holistically are classified or understood to be fragile while in other countries, fragility and conflict is mainly contained to subnational geographies. Thus while South Sudan and Somalia may be classified more generally as fragile due to wider spread conflict and insecurity, in Nigeria and DRC, there are specific regions or states in the country that meet similar criteria. Please see S1Table2 for criteria used to compare the study settings. The data also reflects the primary studies, where the focus in DRC was the Kivu provinces with some interviews with national stakeholders, whereas in Nigeria only state actors were targeted. Thus we acknowledge this limitation in the Limitations section, please see lines 695 to 697.  Our analysis was deductive and focused only on factors related to political prioritization according to the established framework. The original studies had broader sets of questions and analyzed the data differently. However, as shown in S2 Table 4, our sub-themes were created inductively, and added possibly more to the framework per political priority factor. In the original framework, there are no predefined sub-themes for each factor, just a description of what it generally entails. For example, while policy community cohesion is about “degree of coalescence” our coding revealed three sub-themes of NGO actor power and relationships, research and evidence networks and quality of collaborations. Thus our findings don’t diverge from the framework but added more nuance to it.  The supplemental tables S1 Table 2 and S2 Table 4 are added under Supporting Information. |
| The paper explicitly states the focus is on political prioritization rather than prioritization in general (Line 108), but then at the beginning of the Findings it describes “influencing prioritization of MNH in the four settings” (Line 202). It would be helpful to be really clear about the distinction between the two and which is consistently the focus of the paper throughout. | This was an omission and we have specified political prioritization in line 213. The definition is that contained from line 117 through 130. The rest of the paper was checked to ensure we always refer to political prioritization. |
| A couple of more minor points: Some of the quotes are very long, and could potentially be cut down to make the same point and ensure the reader is clear about the purpose of the quote. Line 120 – last box of table, incomplete sentence? “May address”…MNH? Line 281 – MNH or MNCH? If MNCH spell out as don’t think you mentioned before. Line 349 – “regarded AS essential” Line 350 – what kind of politics? There are a few times politics is mentioned, but is a very broad term, would be helpful to have more specificity. Line 459 – “one of THE factors” | We have reviewed the quotes and have shortened some please see lines 240-242; line 282 (removed repetitive quote); line 355; 377-381; 495-500; 541-544.  Line 120 – now line 129 and edited “Stakeholder perspectives of conditions for effectiveness, provider capacity, quality of care (including feedback mechanisms) and how their implementation may address maternal and newborn morbidity and mortality”  Line 281 – now line 298 spelled out in full maternal newborn and child healthcare units.  Line 349 – now line 367, edited.  Line 350 – now line 368, have specified bureaucratic politics and added a reference. Line 42 now specifies actor and budget politics.  Line 459 – now line 488, edited. |
| Reviewer #2 | |
| Thank you for this important work. More attention is needed to healthcare delivery, policy and implementation - and this paper really helps to understand the context based solutions better.  My comments - all minor - were to improve the strength of presentation and make the paper more methodologically stronger just by explaining a bit more. | Thank you for your comments, they are indeed helpful |
| Overall: Based on recent critiques from scholars in the African region, I encourage authors to move away from using “Sub-Saharan Africa” given its colonial roots and non-evidence informed terminology. Since the authors have used a mix of “Africa,” “sub-Saharan Africa,” and “African region” – I think it will be easier to remove sub-Saharan Africa without compromising the clarity or quality of the paper. Please consider revising the term after considering these sources: <https://www.devex.com/news/opinion-why-we-ve-stopped-using-the-term-sub-saharan-africa-105692> (same piece without a paywall: <https://www.firelightfoundation.org/blog/why-weve-stopped-using-the-term-sub-saharan-africa>) <https://www.africarebirth.com/the-racist-connotations-of-the-term-sub-saharan-africa/> | We agree with the sources and the current discourse and have standardized to Africa in this paper. |
| Methodology: It would be good to say that qualitative analysis was “deductive analysis” since the authors used a framework and established themes to group data. <https://researchmethodscommunity.sagepub.com/blog/qualitative-research-design-and-data-analysis-deductive-and-inductive-approaches> | Indeed our approach was deductive. Please see line 177. |
| Results: Since the results section is quite long, would be good to give an overview at the top outlining the themes and what are within. For example, leadership theme under Actor power has multiple themes including gender equity. Highlighting these themes, subthemes at the beginning maybe in a summary table perhaps will be helpful to the reader – also to use this paper for policymaking approaches. Alternatively, a prose outline of the results can be provided at the beginning of the results section, and a summary table can be provided at the end of results. Summary table can be easily drafted using main sections and subthemes. I recognize that there is a detailed supplementary table (S2 Table 4), I wonder if this can be repurposed and included in the manuscript. I think it will greatly improve the presentation of results. | A prose outline of the results is now provided at the beginning, as well as a summary table. Please see line 213-222 for the prose and 225 to 227 for the table. |
| Line 462 – might be good to also include a quote from DRC if one is readily available, since DRC is mentioned. It might be good cut down the South Sudan quote a bit, if possible.  This is not a must – but across the results section, when possible, I would cut down on long quotes – without compromising the quality of results. I think some repetition could be reduced. I leave it to the discretion of the authors and their stylistic approach of how they envision to present the results. Generally, readability would improve if you can cut down repetition within quotes, where possible. | For brevity of the this section, we have shortened ( line 494 to 500) the South Sudan quote.  We have edited the long quotes and removed those that are similar to another, please see lines 240-242; line 282 (removed repetitive quote); line 355; 377-381; 495-500; 541-544. Please also see our responses above to a similar comment from Reviewer #1. |

We thank you for your insightful inputs.

Yours sincerely

Mamothena Mothupi
